# Comparing NIRS and Pulse Oximetry for Cerebral Oxygen Saturation During Hypoxia Testing

**DOI:** 10.3390/medsci12040059

**Published:** 2024-10-24

**Authors:** Vasilios Alevizakos, Andreas Werner, Lisa-Marie Schiller, Constantin von See, Marcus Schiller

**Affiliations:** 1 Research Centre for Digital Technologies in Dentistry and CAD/CAM at Danube Private University, 3500 Krems an der Donau, Austria; constantin.see@dp-uni.ac.at; 2 Flight Physiological Diagnostics and Research, Center for Aerospace Medicine, 10117 Berlin, Germany; andreas4werner@bundeswehr.org; 3 Institute of Occupational, Social, and Environmental Medicine, RWTH Aachen, 52074 Aachen, Germany; 4 Institute of Physiology and Center for Space Medicine and Extreme Environments, Charité University Medicine, 10117 Berlin, Germany; 5 Clinic for Conservative Dentistry, Periodontology and Preventive Dentistry at Hannover Medical School, 30625 Hanover, Germany; lisa-marie.schiller@gmx.net; 6 Department of Oral and Maxillofacial Surgery, Hannover Medical School, 30625 Hanover, Germany; marcus_schiller@yahoo.com

**Keywords:** cerebral oximetry, near-infrared spectroscopy (NIRS), pulse oximetry (SpO2), hypoxia testing, aviation safety, oxygen saturation monitoring

## Abstract

**Objective:** This study evaluates the suitability of cerebral oximetry using near-infrared spectroscopy (NIRS) compared to traditional pulse oximetry (SpO2) for measuring cerebral oxygen saturation during hypoxia testing, aiming to enhance safety during flight operations and training. **Material and Methods:** The study included 106 participants aged 18–60 years at the Aerospace Medicine Training Center in Königsbrück. Cerebral oxygen saturation (rSO2) and peripheral oxygen saturation (SpO2) were measured using the INVOS™ 5100C cerebral oximeter and Masimo™ MS5 pulse oximeter, respectively. Measurements were taken at baseline, during hypoxia at 25,000 feet, and post recovery. Data analysis included regression analysis, Bland–Altman plots, and concordance correlation coefficients (CCC). Ethical approval was obtained from the Hannover Medical School. Data from 100 participants were analyzed. **Results:** Baseline SpO2 was 97.5 ± 1.5%, and baseline rSO2 was 77.25 ± 6.4%. During hypoxia, SpO2 dropped significantly, while rSO2 showed higher values. SpO2 recovered faster than rSO2. Deviations in rSO2 between the right and left sides during hypoxia were minimal. Lin’s CCC indicated moderate to substantial concordance. NIRS measurements were more stable and less prone to disturbances, with 95 disruptions in pulse oximetry, 25 of which were potentially critical. **Conclusions:** NIRS is a reliable method for detecting cerebral oxygen saturation, offering significant advantages over traditional pulse oximetry in stability and reliability during hypoxia testing. NIRS is less error-prone, supporting its use for continuous monitoring in aviation settings and enhancing flight safety by providing more accurate hypoxia detection.

## 1. Introduction

The Wright brothers etched their names in history in 1903 with the inaugural flight of their powered airplane, the “Flyer I”. Despite its modest achievement of hovering just a few meters above the ground for a brief 12 s, this momentous event is widely hailed as the dawn of powered aviation [1,2]. With the rapid evolution of aviation technology, pilots have faced increasing demands due to lengthier flights, higher altitudes, and more sophisticated aircraft systems.

One significant risk to flight personnel is altitude-induced oxygen deficiency. In the U.S. Navy and U.S. Air Force, 221 hypoxia incidents were recorded over 25 years due to cabin pressure drops [3]. A notable series of hypoxia-like events in 2017 involving F-18 Hornet, F-22 Raptor, and F-35 jets in Arizona prompted a thorough investigation. Prior to these incidents, anonymous U.S. pilots had already voiced concerns about hypoxia, indicating that while the issue was acknowledged, prevention measures were insufficient. Each flight puts pilots in a precarious position, relying on technology to safeguard their lives and on robust preventative measures to mitigate risks like hypoxia.

The human body typically operates under stable conditions, maintaining oxygen levels through breathing and blood circulation. Hemoglobin in red blood cells binds and transports oxygen to organs. However, deviations from these conditions require the body to adapt, as seen when climbing a high mountain. Pilots must similarly adjust to rapidly changing altitudes. Diverse factors can lead to oxygen deficiency in an aircraft, such as cabin pressure drops from intentional or accidental openings, technical failures, or electrical fires. Pilots must recognize their unique hypoxia symptoms and act swiftly if control and warning systems fail [4,5,6,7,8]. Hypoxia, the insufficient supply of oxygen to body cells, can escalate to anoxia, leading to death [9]. Recognizing hypoxia symptoms is crucial, as they can vary greatly. Common symptoms include temperature dysesthesia, dizziness, tingling, nausea, or impaired vision [4,6,7,10]. These symptoms can progress to incapacitation, with effective performance time (EPT) rapidly decreasing at higher altitudes [7]. For instance, at 18,000 feet, a pilot has about 30 min of EPT, but this shrinks to 30–60 s at 35,000 feet, and mere seconds at 38,000 feet. Time of useful consciousness (TUC) represents the active period of functionality, which can be further reduced by factors like physical activity and smoking [7,9,11].

Early recognition of hypoxic conditions is vital, relying heavily on the pilot’s experience and situational awareness [7]. Various factors, such as engine exhaust ingestion leading to carbon monoxide poisoning or circulatory issues, can complicate oxygen deficiency. Excessive alcohol consumption, acting as a cellular toxin, can also impede oxygen uptake.

**Figure 1:**
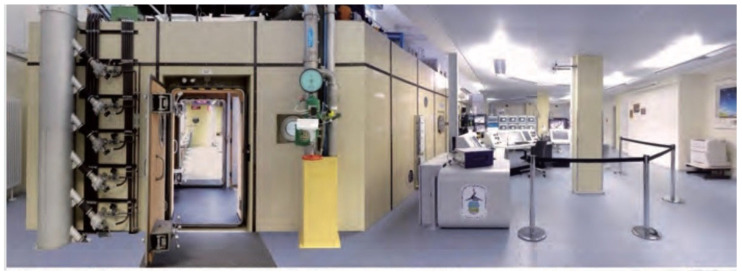
Altitude–climate situation chamber in Königsbrück, with a view of the control station and into the chamber (Source: Senior physician PD Dr. Carla Ledderhos).

In the German Armed Forces, pilots and flight personnel, including AirMedEvac crews and high-altitude parachutists, undergo hypoxia symptom training every four years at the Aerospace Medicine Training Center in Königsbrück. This training involves simulated high altitudes in a hypobaric chamber, where participants learn to recognize their hypoxia symptoms and effectively use their EPT or TUC to respond to initial symptoms by regulating oxygen or reducing altitude (Figure 1).

The development of oximetry has been crucial in monitoring oxygen levels. First described by Nicolai in 1932 and refined by Matthes in 1935, oximetry technology advanced significantly during World War II and saw renewed interest in the 1970s and 1980s [12,13,14]. Modern pulse oximeters, small devices typically attached to a fingertip, earlobe, or toe, measure oxygen saturation using light absorption principles [15,16]. Reflectance oximeters, used in fitness wearables, detect reflected light and can be attached to the forehead.

**Figure 2:**
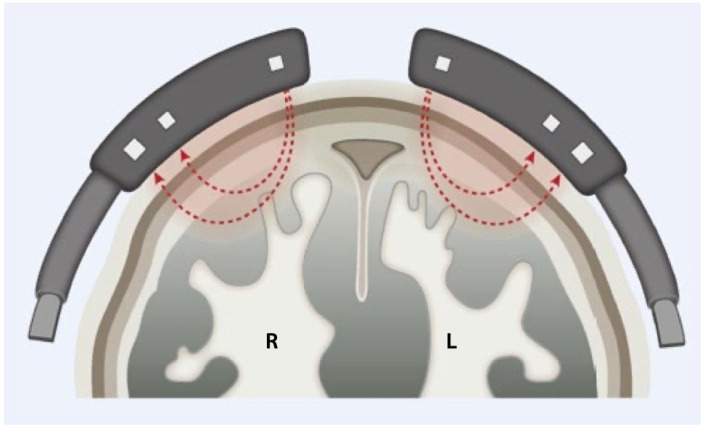
Schematic representation of cerebral oximetry. L = left; R = right (Source: Courtesy of Springer Nature).

Cerebral oximetry, like pulse oximetry, measures oxygen saturation through the skull using light absorption and scattering principles (Figure 2) [17]. Commercial systems like those by near-infrared spectroscopy (NIRS) or in vivo optical spectroscopy (INVOS) are widely used, including by athletes to measure muscle oxygenation [17,18,19,20].

These monitoring technologies are essential for identifying hypoxia and enhancing flight safety and performance, especially during high-altitude and long-duration missions. Recognizing hypoxia symptoms and utilizing advanced monitoring tools significantly contribute to the safety and efficiency of modern aviation.

The aim of the study is to evaluate the suitability of cerebral oximetry using NIRS compared to the traditional SpO2 measurement and to assess any deviations between these two measurements under hypoxic and normoxic conditions.

## 2. Materials and Methods

The clinical study application (Number: 9214_BO_K_2020) was approved by the Ethics Committee of the Hannover Medical School as of 7 July 2020. This approval also considered an existing ethics vote from Charité dated 30 March 2017 (EA1/023/17).

The study was conducted with 106 training participants (TP) who participated in regular chamber flights at the high-altitude climate simulation system (HKS) in Königsbrück as part of their flight physiological training. Participants of both genders aged between 18 and 60 years were included. Prior to the study, participants were thoroughly informed about the study and its voluntary nature. Each participant could withdraw from the study at any time. They were also informed that participation or non-participation would not affect their training course or any official matters.

The fundamental requirement for participation in the chamber flight is a valid flight medical certificate (military pilot fitness (WFV)), issued annually by the Center for Aerospace Medicine of the German Air Force following a comprehensive medical examination. An additional exclusion criterion for study participation was a plaster allergy.

The study was conducted at the HKS of the Center for Aerospace Medicine of the German Air Force in Königsbrück. Between June and November 2017, 106 participants were monitored using cerebral oximetry during 53 chamber flights. Up to two of a maximum of six participants per chamber flight were additionally monitored with cerebral oximetry alongside standard monitoring (ECG, heart rate (HR); respiratory rate (RR), SpO2).

Before the chamber flight, participants were questioned about the following: age, height, handedness, smoking status, current medication use, number of chamber flights, flight hours, aircraft types, and nationality.

Before and after the chamber flight, the following parameters were measured: weight, body impedance analysis (BIA), and blood pressure (BP) via Riva–Rocci (RR), HR, and SpO2. During the chamber flight, ECG was recorded, and pulse, RR, SpO2, and regional cerebral oxygen saturation (rSO2) were measured.

The pulse oximeter used was the Masimo™ MS5 module (GE Healthcare, Chicago, IL, USA). It measures SpO2 and peripheral pulse, with saturation ranging from 1–100% in 0.1% increments. The device has a root mean square deviation (RMSD) of 2% for SpO2 values between 70–100%, 3% for values between 50–69%, and 4% for values below 50%. This statistical accuracy indicates that approximately two-thirds of the measurement values fall within 2% of the reference blood gas values, which is typical for most commercial pulse oximeters. Peripheral pulse is displayed in the range of 25–240/min with an accuracy of 4 beats per minute in the range of 30–199/min, with averaging options over 4, 8, or 16 s, and a measurement is stored every 2 s (manufacturer’s specifications).

Among the available sensors, the finger clip was used. The normal value is 97–99%. The TP wore the finger clip on their left hand, as the right hand was used for operating the control stick.

The cerebral oximeter used was the INVOS™ 5100C (Medtronic, Dublin, Irland). It utilizes spatially resolved continuous-wave technology at two near-infrared wavelengths of 730 and 810 nm, allowing for the measurement of absolute values rather than relative changes, as seen in other standard CW NIRS devices. A self-adhesive sensor, the SomaSensor^®^ for adults, was placed on the forehead paramedian below the hairline and above the eyebrows. It consists of a light-emitting diode and two detectors (flat and deep detector) in a light-tight adhesive patch. rSO2 is displayed in the range of 15–95%. The manufacturer specifies hardware repeatability with 1 rSO2 index point from unit to unit, including the SomaSensor^®^. Data are recorded every 5–6 s. According to Davie et al., the INVOS 5100 used showed an rSO2 value of 76.6 ± 9.6% in young healthy patients [20].

The manufacturer specifies environmental limitations, including a maximum altitude of 10,000 ft. Therefore, the monitoring unit had to remain outside the chamber. A shielded 1.5 m cable connected the SomaSensor^®^ to the preamplifier, and a second shielded 4.5 m cable connected the preamplifier to the monitoring unit. The preamplifier cable was severed and passed through the chamber wall so that the monitor could remain outside.

Participants were investigated according to a standardized protocol. Up to six training participants could be included in each chamber flight. An observer (internal companion (IC)) was present at each flight for vital monitoring of the participants and had continuous access to 100% O2 for use if needed.

A preliminary 30 min oxygen pre-breathing phase at ground level (175 m above sea level) was performed to prevent decompression sickness (DCS) and to eliminate approximately 30% of nitrogen from the body.

This was followed by a test ascent to 8000 ft (rate v = 4000 ft/min) with subsequent pressure increase back to 3000 ft (v = 2000 ft/min) to ensure smooth sinus pressure equalization via the Eustachian tube. The actual ascent to 25,000 ft (v = 4000 ft/min) then occurred. At this plateau, a hypoxia simulation was performed by decreasing FiO2 from 100% to 21%, allowing participants to identify their individual hypoxia symptoms within a maximum of 5 min.

If a participant identified two symptoms of hypoxia or experienced a severe symptom (e.g., dizziness), the hypoxia simulation was terminated by reintroducing pure O2. If a participant’s SpO2 reached 70% without symptoms, O2 was reintroduced either by the participant, the IC, or the aerospace physiology officer (APO) to prevent incapacitation or unconsciousness.

Following this, the pressure was decreased to 15,000 ft (v = −4000 ft/min), corresponding to the second training altitude. At this level, O2 was reduced back to FiO2 21% for all participants (mild hypoxia demonstration—mSMD). At this simulated altitude, color vision disturbances and psychological tests for mental retardation were conducted either after SpO2 dropped below 90% in all participants or after a maximum of 3 min. The maximum duration without SpO2 was 12 min, after which 100% O2 was administered.

**Figure 3:**
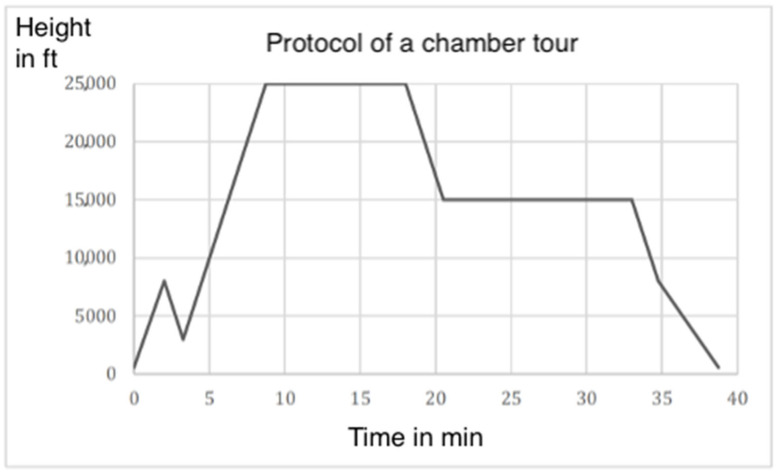
Example of a protocol of a chamber tour (Source: Senior physician Dr. Werner).

The chamber pressure was then reduced at a rate of −4000 ft/min to 8000 ft. From 10,000 ft, the chamber pressure was adjusted to ambient air and then reduced at −2000 ft/min back to ground level (Figure 3).

Consultation was provided by the Institute for Biometry and Epidemiology at Charité, University Medicine Berlin. Statistical methods included regression analysis and the concordance correlation coefficient. Based on a significance level of α = 0.05 and a power of 80%, the required sample size was determined to be 60.

Method comparison was performed using regression analysis and depicted according to Bland–Altman and the concordance correlation coefficient (CCC) according to L.I. Lin. Mean (MW) and standard deviation (SD) were reported, and the Wilcoxon signed-rank test for paired samples was used, with significance set at *p* < 0.05.

The collected data for SpO2 and HR from the HKS were directly transferred to Excel 2013 (Microsoft, Redmond, WA, USA). The data from the INVOS™ 5100C oximeter were processed with the analysis software “Shortcut to INVOS™ -analytics tool” (Covidien, Dublin, Ireland) and then exported to an Excel spreadsheet.

For general statistical processing, SPSS Statistics 20 (IBM, Armonk, New York, NY, USA) was used. CCC calculation was performed using an online calculator (https://www.statstodo.com/LinCCC.php, accessed 3 July 2018).

## 3. Results

Out of the 53 chamber flights conducted, data from the HKS could not be saved and reconstructed for four participants. Additionally, there were two faulty measurements with the NIRS: In one case, INVOSTM data were only recorded for mSMD, and in another case, cerebral oxygen saturation was measured unilaterally. Consequently, these six datasets were excluded, resulting in 100 complete datasets included in this study.

Participants’ ages ranged from 22 to 59 years, with a median age of 34 years, a 10th percentile of 27 years, and a 90th percentile of 45 years. The gender distribution consisted of 9 female and 91 male participants. The sample size for female participants (*n* = 9) is too small for subgroup analysis.

Vital signs were routinely recorded before and after the chamber flight, as detailed in Table 1.

Participants were also surveyed about their subjective symptoms during oxygen deprivation. The most common symptoms reported were warmth, shortness of breath, and dizziness, followed by tingling, rapid pulse, changes in vision, and pressure sensations in the head and chest. The distribution of acute hypoxia symptoms is detailed in Table 2.

Moreover, 60% of participants reported only two symptoms, while 16% reported three. After the aSMD, 80% of participants indicated additional symptoms. On average, symptoms were reported after 103 s with a peripheral saturation of 87%. The first participant reported symptoms after 57 s, while the last participant did so after 192 s with an SpO2 of 71%. After mSMD, only qualitative changes in symptoms were recorded, with 56 participants reporting physical symptoms.

Peripheral and cerebral saturation levels were measured before, during, and after hypoxia. The baseline values at 570 ft are presented in Table 3.

In Table 3, we observe that SpO2 values demonstrate a narrow range, indicating more consistent readings across subjects. In contrast, P0 rSO2 (NIRS) values present a wider range of variability, both for the right and left sides. This variability could reflect inter-subject differences, which may arise from factors like tissue composition, probe placement, and physiological responses under hypoxic conditions.

Such inter-subject variability in NIRS measurements may impact the reliability of using NIRS as a standalone tool for monitoring hypoxia, particularly in high-performance settings like pilot training. While NIRS provides valuable information about regional oxygenation, the broader range of values suggests that thresholds for hypoxia detection may not be as easily defined or applied uniformly across individuals. This could lead to challenges in standardizing its use in training environments where precise and reliable detection of hypoxia is critical. Further research may be needed to explore how these variability patterns affect clinical decision making and whether calibration methods or combined metrics (such as integrating NIRS with SpO2) might improve monitoring accuracy.

The peripheral saturation at sea level was 97.5 ± 1.5%, which is within the known normal range of 97–99%. The measured cerebral saturation was 77.25 ± 6.4%, which is in the upper expected range. It has been reported an average rSO2 of 71 ± 6% in young, healthy volunteers [21].

Values before hypoxia at 25,000 ft (P1) are detailed in Table 4.

The lowest saturation values at 25,000 ft (P2) are shown in Table 5.

After the recovery of the participants at 25,000 ft, the values recorded (P3) are listed in Table 6.

The recovery of the initial values of both SpO2 and rSO2 is recognizable.

**Figure 4:**
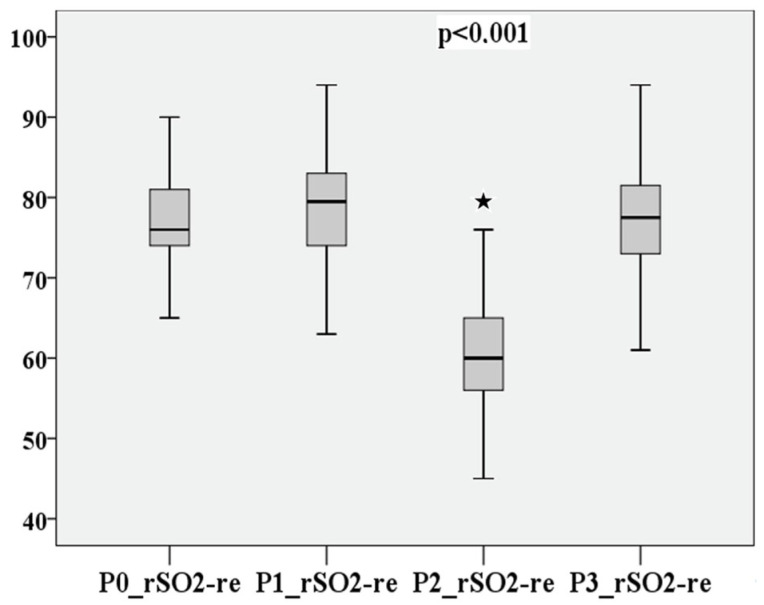
rSO2 on the right at measurement points P0–P3.

**Figure 5:**
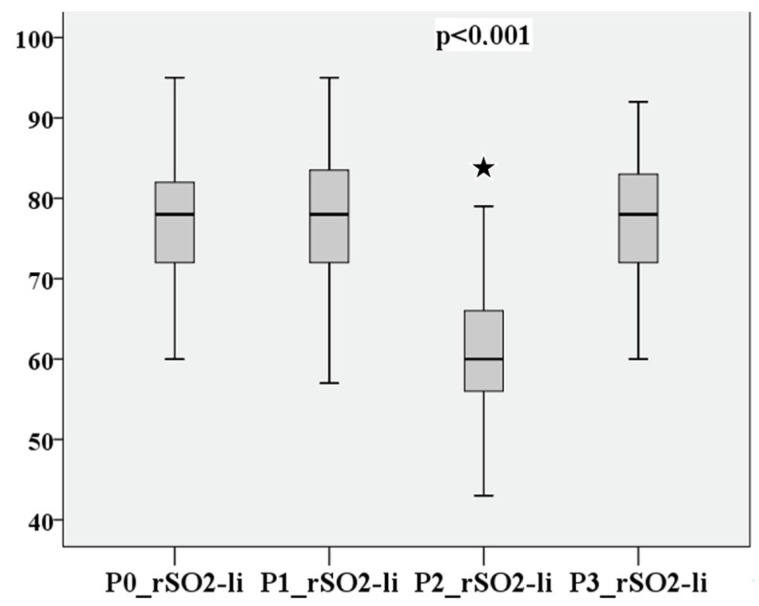
rSO2 on the left at measurement points P0–P3.

P0–P3 of cerebral saturation on the right and left are compared in Figure 4 and Figure 5.

**Figure 6:**
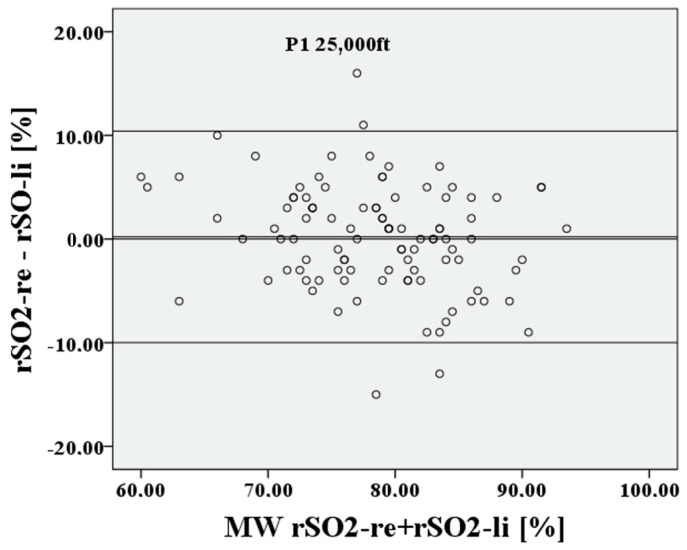
Relationship of rSO2-right to rSO2-left, based on the mean at the lowest saturation P1.

**Figure 7:**
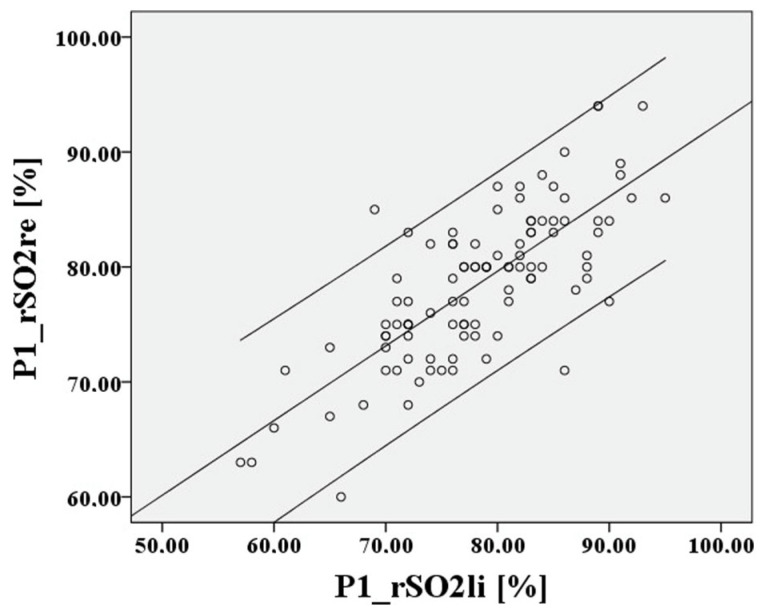
Relationship of rSO2-right to rSO2-left at the lowest saturation P1.

Subsequently, for the value P1, i.e., before hypoxia, a Bland–Altman diagram is shown in Figure 6 and Figure 7.

**Figure 8:**
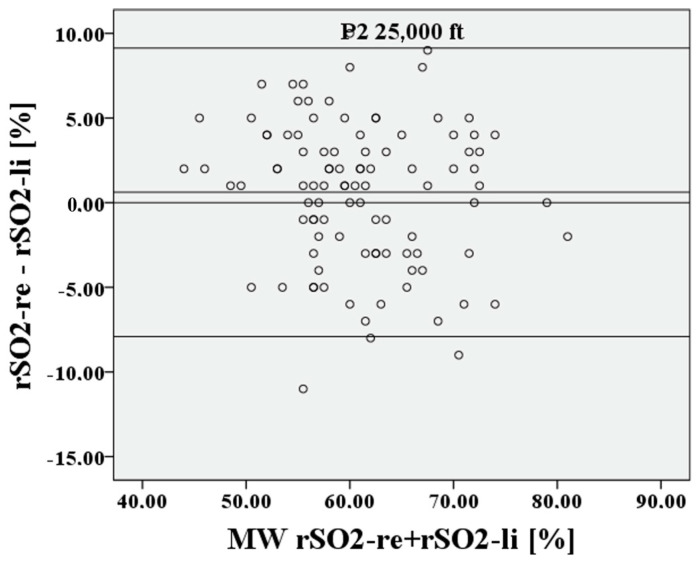
Relationship of rSO2-right to rSO2-left, based on the mean at the lowest saturation P2.

**Figure 9:**
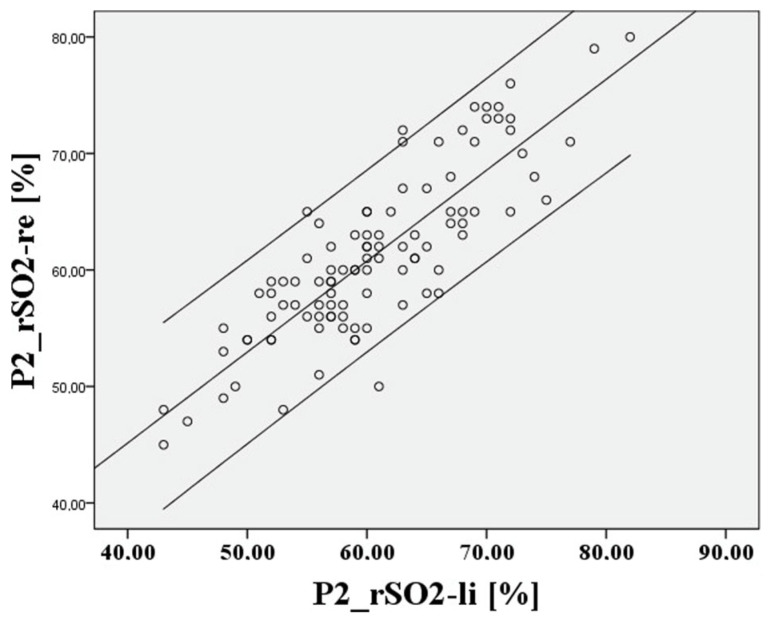
Relationship of rSO2-right to rSO2-left at the lowest saturation P2.

The deviation of the rSO2 measurements between right to left is approximately 0.21 percentage points, the 95% confidence interval lies between 10.41% and −9.99%, the limits of agreement (LoA) are clinically acceptable at 10.2%, and Lin’s concordance correlation coefficient (CCC) is moderate at 0.753. For P2, cerebral saturation on the right and left is also represented in Figure 8 and Figure 9 using a Bland–Altman diagram.

**Figure 10:**
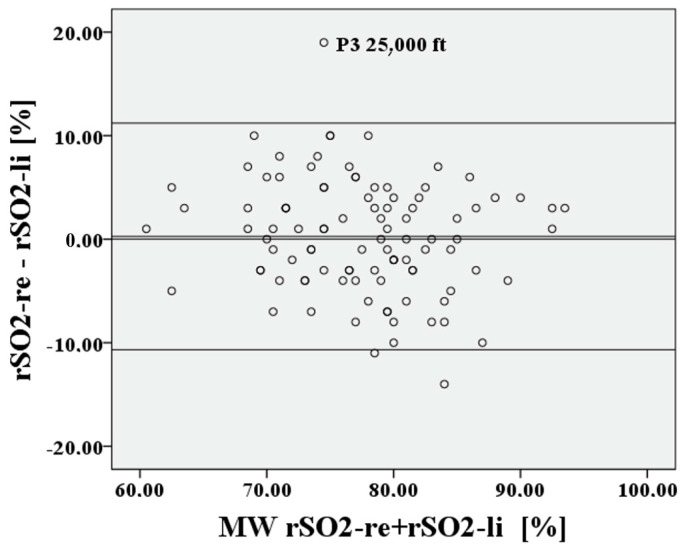
Relationship of rSO2-right to rSO2-left, based on the mean after hypoxia P3.

**Figure 11:**
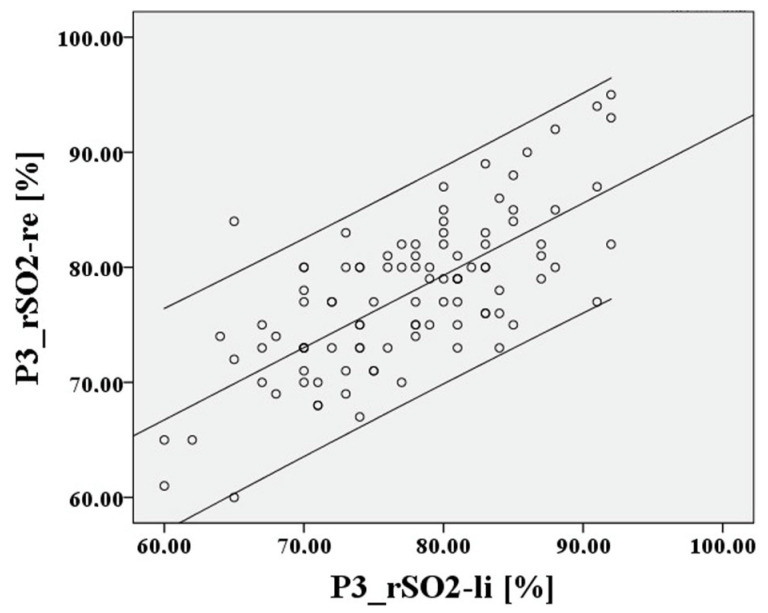
Relationship of rSO2-right to rSO2-left after hypoxia P3.

The deviation of the rSO2 measurements between right to left during hypoxia is extremely small at 0.62 percentage points, the 95% confidence interval lies between 9.14% and −7.9%, the LoA are again acceptable at 8.52%, and Lin’s CCC is substantial at 0.838. After recovery at P3, the result is like P1. This is shown in Figure 10 and Figure 11.

The deviation of the rSO2 measurements between right to left is extremely small at 0.27 percentage points, the 95% confidence interval lies between 11.21% and −10.67%, the LoA are 10.94%, and Lin’s CCC is moderate at 0.701.

**Figure 12:**
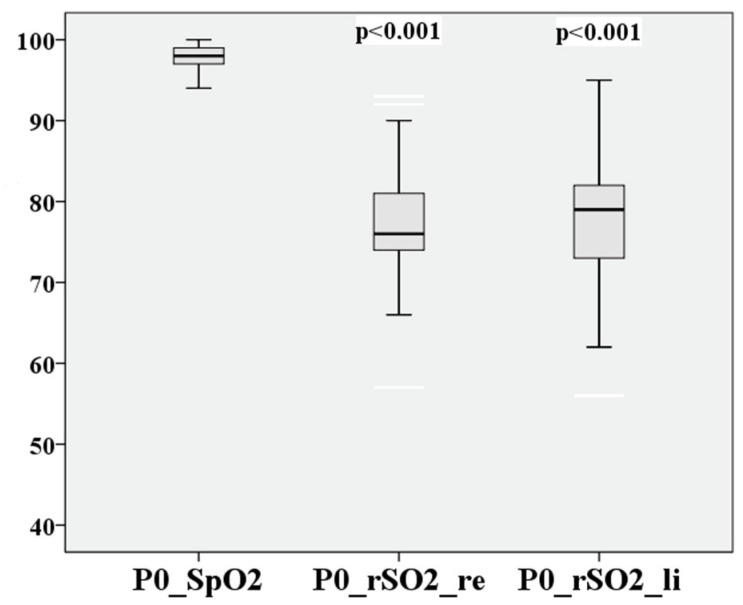
Comparison of peripheral and cerebral oxygen saturation of the baseline value (P0).

**Figure 13:**
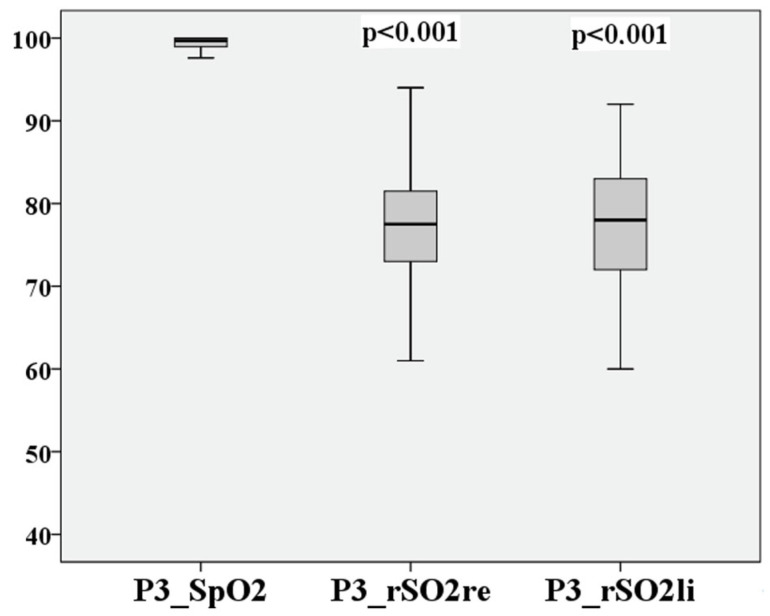
Comparison of peripheral and cerebral oxygen saturation before hypoxia (P1).

**Figure 14:**
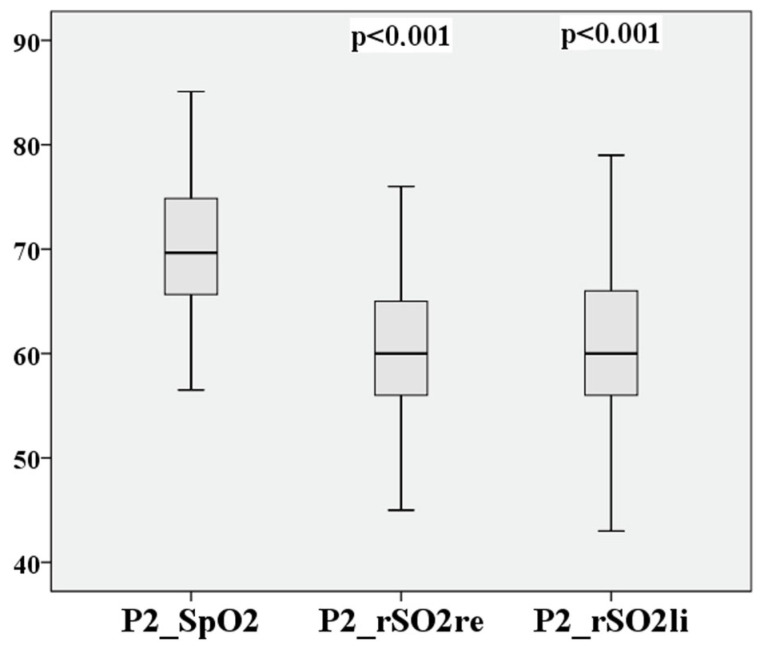
Comparison of peripheral and cerebral oxygen saturation at the lowest saturation value (P2).

**Figure 15:**
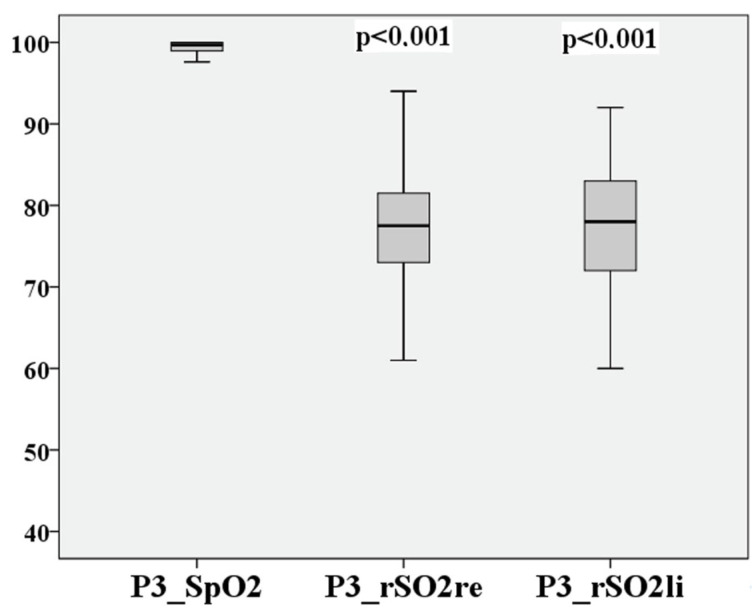
Comparison of peripheral and cerebral oxygen saturation after hypoxia (P3).

The measurement points P0–P3 are directly compared in Figure 12, Figure 13, Figure 14 and Figure 15 for cerebral and peripheral measurements to graphically represent the comparability of the two measurement methods. The difference in rSO2 and SpO2 is highly significant at each of the defined time points (*p* < 0.01).

From the time of the start of oxygen deprivation demonstration at P1 and the time of the lowest saturation P2, T1 can be determined, subsequently leading to recovery saturation at P3, resulting in T2 (Table 7 and Table 8).

The tolerance of the subjects until intervention shows a 95% confidence interval around the mean of approximately 100 s, ranging from 40 to 160 s.

The recovery time in the arterial compartment is shorter at 25.88 ± 11.31 s. In the venous-weighted compartment, the recovery time is more than twice as long, with a mean of 58.47 ± 23.7 s.

## 4. Discussion

In this study, it was found that NIRS consistently recorded stable rSO2 values during hypoxic demonstrations, with significant decreases observed under hypoxia. These findings align with the research conducted by Smith et al., who emphasized that cerebral oxygenation is critically impacted by hypoxia [8]. Their work highlighted the importance of continuous monitoring in high-risk environments, such as military aviation, where sudden changes in oxygen availability can occur. Their findings underline the relevance of this study in improving safety measures for military personnel during flight operations.

Furthermore, the distinction between pulse oximetry and NIRS measurements is crucial. As noted in the work of Torp et al., pulse oximetry primarily reflects arterial oxygen supply, while NIRS provides a venous-weighted assessment that balances oxygen supply and demand [20]. This fundamental difference is essential for interpreting the results, particularly in the context of hypoxic conditions. For instance, while pulse oximetry showed stable SpO2 during baseline conditions, its values dropped significantly under hypoxia. In contrast, NIRS effectively captured the reduction in cerebral oxygenation, reinforcing the notion that relying solely on SpO2 may not adequately reflect tissue oxygenation status during critical situations. The implications of these measurement differences are particularly pertinent in military settings, where understanding the physiological state of pilots can be vital for mission success and safety.

The findings regarding the impact of body mass index (BMI) on NIRS measurements further corroborate the literature. Beever et al. reported that soldiers, particularly aviators, may have higher BMI due to increased muscle mass rather than fat [22]. This suggests that BMI may not significantly affect NIRS readings, as no notable differences in rSO2 values were observed among participants with varying BMI. This aligns with the premise that active individuals may maintain better tissue oxygenation than sedentary populations, even at elevated BMI levels.

Additionally, the physiological implications of tissue oxygenation deficits following the restoration of oxygen supply are significant. Research underscores that tissue oxygenation may not immediately recover after hypoxic exposure, emphasizing the importance of continuous monitoring for identifying ongoing oxygen deprivation [23]. The study’s findings—that tissue oxygen deficiency can persist even when oxygen supply is restored—highlight the advantages of rSO2 in detecting tissue hypoxia compared to SpO2, which reflects only oxygen availability. This information could be critical for military personnel, where understanding physiological responses to hypoxia could influence operational strategies and training protocols.

Moreover, the investigation into the effects of smoking on pulse oximetry readings adds another layer to the discussion. While baseline SpO2 values were comparable between smokers and non-smokers among the participants, the implications of reduced peripheral circulation in smokers are well documented in the literature. The work of Glass et al. demonstrated that smoking can significantly impair peripheral circulation, potentially skewing pulse oximetry readings [24]. However, the results suggest that even within this population, pulse oximetry maintained a level of reliability, further supporting the need for complementary monitoring techniques such as NIRS in situations where traditional methods might face limitations.

Integrating these studies into the discussion aims to create a comprehensive contextual framework for the findings. This approach reinforces the significance of the results and contributes to the ongoing discourse on non-invasive monitoring techniques in aviation and military contexts. The evidence suggests that understanding tissue oxygenation under hypoxic conditions is critical for enhancing the safety and performance of military aviation personnel.

Based on consultations with the Institute for Biometry and Epidemiology of Charité—Universitätsmedizin Berlin, the required sample size of 60 subjects was calculated. To ensure robust results, it was necessary to account for the potential dropout rate in the study. Consequently, 106 participants were selected for the investigation, with 100 ultimately included in the analysis. The primary goal of this study was to compare two different non-invasive methods for determining tissue oxygenation in military aviation personnel. The secondary aim was to enhance safety during flight operations and training based on the findings. A thorough understanding of tissue hypoxia and its detection methods is critical for improving operational readiness in aviation contexts, particularly in ensuring that pilots are equipped with the right tools and trained effectively to recognize and respond to hypoxic conditions.

To investigate SpO2 in relation to hypoxia symptoms, it was essential to use a method capable of providing oxygen saturation values at frequent intervals without interfering with the monitored personnel’s duties. Pulse oximetry, a long-established method for monitoring SpO2, was employed in this study. The pulse oximeter was attached to the left hand of participants, which is particularly relevant for jet pilots, as the left hand is primarily used for operating buttons and checking the oxygen mask fit. While movement artifacts were inevitable, these were consistently accounted for in the analysis. Notably, the pulse oximeter exhibited sensitivity to movement-induced disturbances, with instances where no value was displayed and error messages such as “loose” or “search” appeared, potentially delaying monitoring by up to 8 s. Such delays could have serious implications for pilots during critical flight operations, emphasizing the need for reliable monitoring systems. This aligns with the findings of Petterson et al., who also noted that movement artifacts can significantly affect the accuracy of pulse oximetry in dynamic environments [25].

Alternative pulse oximetry sensors, such as adhesive ear or nasal sensors, may reduce displacement issues; however, helmet displacement of ear sensors or mask-related issues with adhesive nasal sensors must be considered. Temperature fluctuations due to varying altitude profiles pose another challenge, as cooling in the chamber can reduce peripheral circulation, affecting SpO2 measurement accuracy. This finding is supported by the work of Ascha et al., which highlighted the challenges of maintaining accurate oxygen saturation readings in varying environmental conditions, particularly in aviation settings [26].

In contrast, NIRS has gained attention for its non-invasive capabilities and was chosen as a comparative measurement method due to its extensive clinical experience. Throughout the study, the NIRS device provided accurate readings, with no disturbances observed in the 100 participants. The forehead-attached sensor demonstrated robustness against movements and vibrations, indicating that NIRS is a practical method for continuous monitoring in aviation environments. This reinforces the findings of Zhou et al., which documented the effectiveness of NIRS in stable and dynamic settings [27].

In the investigation, NIRS consistently recorded stable rSO2 values across all participants, reflecting its potential for real-time monitoring of pilots’ tissue oxygenation. The average rSO2 values during baseline conditions were 77.25% ± 6.41 for the right side of the brain and 77.51% ± 7.43 for the left side, which decreased significantly to 61.26% ± 7.25 and 60.64% ± 7.83, respectively, during the hypoxic state. In contrast, pulse oximetry provided stable SpO2 values at baseline, averaging 97.5% ± 1.5, but showed a notable decline to 69.97% ± 6.67 during hypoxia. The difference between the two measurement techniques illustrates the importance of understanding the physiological implications of tissue oxygenation. While pulse oximetry measures arterial oxygen supply, NIRS provides a venous-weighted assessment, offering a more comprehensive understanding of how tissue responds under hypoxic conditions. This differentiation aligns with research by Cheung et al., who emphasized the relevance of combining both measurement techniques to gain a full understanding of oxygenation status [28].

Moreover, the study highlighted the influence of BMI on NIRS measurements. Although BMI does not differentiate between fat and muscle mass, soldiers, especially aviators, may present higher BMIs due to increased muscle mass rather than fat, which may mitigate the impact on NIRS readings. This contrasts with the findings of Beever et al., which indicated that increased adiposity could negatively affect peripheral oxygenation readings in some populations [22].

Among the 100 participants, 18 were smokers, known to have reduced peripheral circulation, which could influence pulse oximetry readings. However, baseline SpO2 values were comparable across smokers and non-smokers in the study. These findings support the ongoing discourse in the literature regarding the need for tailored monitoring strategies in different populations to enhance flight safety.

The investigation observed notable discrepancies between pulse oximetry and NIRS in measuring tissue oxygenation under hypoxic conditions. Pulse oximetry primarily reflects arterial oxygenation, while NIRS provides a venous-weighted assessment, highlighting the critical physiological differences between the two methodologies.

Numerous studies have documented the limitations of pulse oximetry in accurately assessing oxygenation during acute hypoxia. For instance, Abraham et al. found that while pulse oximetry remains a reliable tool for measuring SpO2, it can be significantly influenced by peripheral circulation changes, often exacerbated in military aviation environments [29]. This aligns with the findings of this study, where movement artifacts and temperature fluctuations in the hypobaric chamber affected the reliability of SpO2 measurements.

In contrast, NIRS has been widely recognized for its ability to provide continuous and reliable measurements of cerebral oxygenation even in challenging conditions. Ali et al. emphasized the effectiveness of NIRS in both stable and dynamic environments, particularly in situations where traditional methods like pulse oximetry may falter [30]. This aligns with the study, as NIRS consistently demonstrated stable cerebral oxygen saturation values throughout the experimental conditions, making it a valuable tool for monitoring pilots’ tissue oxygenation during flight operations.

Temperature plays a crucial role in NIRS accuracy, particularly in military aviation settings, where rapid altitude changes can lead to significant temperature fluctuations. Cold environments can reduce peripheral blood flow due to vasoconstriction, potentially impacting NIRS readings. Donadello et al. highlighted that reduced tissue perfusion in cold conditions may lead to an underestimation of rSO2 [31]. Although this study’s hypobaric chamber maintained a controlled environment, cooling effects still influenced peripheral circulation, underscoring the need for consideration of environmental factors.

Despite these challenges, NIRS provided relatively stable readings compared to pulse oximetry, which was more affected by temperature-induced changes in peripheral circulation. However, mitigating strategies—such as heated sensors or protective headgear—should be considered to minimize temperature-related variability in future applications, emphasizing the need to account for environmental factors in ensuring reliable monitoring of pilots.

The physiological implications of tissue oxygenation deficits during hypoxic exposure are profound. Shaw et al. noted that oxygen deprivation could lead to impaired cognitive function, which is critical in high-stakes environments like military aviation [7]. The significant declines in rSO2 during hypoxia observed in the study underscore the importance of early detection and intervention strategies for maintaining pilots’ operational readiness.

Overall, the findings contribute valuable insights into the comparative effectiveness of pulse oximetry and NIRS in measuring tissue oxygenation under hypoxic conditions. By framing the results within the broader context of the existing literature, the study aims to provide a comprehensive understanding of the implications for military aviation personnel and highlight the need for enhanced monitoring strategies to ensure safety and performance in critical situations.

## 5. Conclusions

The NIRS method is a reliable tool for detecting rSO2 values, which are correlated with symptoms of hypoxia, making NIRS fundamentally suitable for monitoring purposes. In our study, NIRS appeared to be less affected by motion artifacts compared to pulse oximetry. However, it is important to note that this result may be influenced using different sensor locations—the forehead for NIRS and a finger clip for pulse oximetry—rather than an inherent advantage of NIRS. The literature indicates that NIRS is also sensitive to motion artifacts, with various techniques proposed to mitigate this. Additionally, while NIRS measurements in our study were free of disturbances, pulse oximetry experienced 95 events, 25 of which were potentially critical. This suggests that in this specific experimental setup, NIRS provided more reliable measurements. Nonetheless, future comparisons should consider using forehead SpO2 sensors to eliminate potential location dependency and further explore the comparative performance of these methods.

## Figures and Tables

**Table 1:** Vital Values of the Participants Before and After the Chamber Flight.

	Systolic Blood Pressure (Millimeters of Mercury (mmHg))	Diastolic Blood Pressure (mmHg)	Pulse (beats/min)	SpO2 (%)
Before HKS				
Mean	144.8	84.1	74.3	97.5
Standard Deviation (SD)	11.9	8.8	12.7	1.5
After HKS				
Mean	136	82.2	66.3	96.5
Standard Deviation (SD)	11.4	8.9	10	1.4

**Table 2:** Oxygen Deficiency Symptoms of the participants.

Symptom	During Acute O2 Deficiency	Additional Information After Mild O2 Deficiency
Breathing	38%	6%
Heart Rate	19%	1%
Heat	56%	26%
Pressure	5%	2%
Tingling	18%	10%
Dizziness	25%	23%
Vision	16%	9%
Miscellaneous	15%	9%

**Table 3:** Baseline Values of the Participants at 570 ft (Local Elevation).

P570 ft (Local Elevation)	n	Minimum %	Maximum %	Average %	SD
pO2 = 207 Millibar (mbar)					
P0 SpO2	100	94	100	97.54	1.47
P0 rSO2 right	100	57	94	77.25	6.41
P0 rSO2 left	100	56	95	77.51	7.43

**Table 4:** Baseline Values Before Hypoxia at 25,000 ft.

25,000 ft	n	Minimum %	Maximum %	Mean %	SD
pO2 = 376 mbar					
P1 SpO2	100	97	100	99.45	0.73
P1 rSO2 right	100	60	94	78.55	6.66
P1 rSO2 left	100	57	95	78.34	7.82

**Table 5:** Values of Lowest Saturation at 25,000 ft.

25,000 ft	n	Minimum %	Maximum %	Mean %	SD
pO2 = 207 mbar					
P2 SpO2	100	56.5	85.1	69.97	6.67
P2 rSO2 right	100	45	80	61.26	7.25
P2 rSO2 left	100	43	82	60.64	7.83

**Table 6:** Values After Recovery at 25,000 ft.

25,000 ft	n	Minimum %	Maximum %	Mean %	SD
pO2 = 376 mbar					
P3 SpO2	100	96.7	100	99.36	0.79
P3 rSO2 right	100	60	95	77.67	6.65
P3 rSO2 left	100	60	92	77.40	7.48

**Table 7:** Duration of Hypoxia at 25,000 ft.

25,000 ft	n	Minimum (Seconds)	Maximum (Seconds)	Mean (Seconds)	Standard Deviation
T1_SpO2	100	46	184	100.47	30.40
T1_rSO2	100	40	177	103.49	29.18

**Table 8:** Duration of Recovery at 25,000 ft.

25,000 ft	N	Minimum (Seconds)	Maximum (Seconds)	Mean (Seconds)	Standard Deviation
T2_SpO2	100	10	64	25.88	11.31
T2_rSO2	100	18	153	58.47	23.70

## Data Availability

The raw data supporting the conclusions of this article will be made available by the authors on request.
